# Structure, Biosynthesis, and Biological Activity of Succinylated Forms of Bacteriocin BacSp222

**DOI:** 10.3390/ijms22126256

**Published:** 2021-06-10

**Authors:** Justyna Śmiałek, Michał Nowakowski, Monika Bzowska, Oliwia Bocheńska, Agnieszka Wlizło, Andrzej Kozik, Grzegorz Dubin, Paweł Mak

**Affiliations:** 1 Department of Analytical Biochemistry, Faculty of Biochemistry, Biophysics and Biotechnology, Jagiellonian University, Gronostajowa 7 St., 30-387 Kraków, Poland; justyna.smialek@doctoral.uj.edu.pl (J.Ś.); oliwia.bochenska@gmail.com (O.B.); agnieszka.wlizlo47@gmail.com (A.W.); andrzej.kozik@uj.edu.pl (A.K.); 2 Biological and Chemical Research Centre, Faculty of Chemistry, University of Warsaw, Zwirki i Wigury 101 St., 02-089 Warszawa, Poland; lyam@chem.uw.edu.pl; 3 Department of Cell Biochemistry, Faculty of Biochemistry, Biophysics and Biotechnology, Jagiellonian University, Gronostajowa 7 St., 30-387 Kraków, Poland; monika.bzowska@uj.edu.pl; 4 Małopolska Centre of Biotechnology, Jagiellonian University, Gronostajowa 7a, 30-387 Kraków, Poland; grzegorz.dubin@uj.edu.pl

**Keywords:** bacteriocin, antibacterial, cytotoxic, *Staphylococcus pseudintermedius*, succinylation, succinyl-coenzyme A, nuclear magnetic resonance (NMR), Krebs/tricarboxylic acids cycle

## Abstract

BacSp222 is a multifunctional peptide produced by *Staphylococcus pseudintermedius* 222. This 50-amino acid long peptide belongs to subclass IId of bacteriocins and forms a four-helix bundle molecule. In addition to bactericidal functions, BacSp222 possesses also features of a virulence factor, manifested in immunomodulatory and cytotoxic activities toward eukaryotic cells. In the present study, we demonstrate that BacSp222 is produced in several post-translationally modified forms, succinylated at the ε-amino group of lysine residues. Such modifications have not been previously described for any bacteriocins. NMR and circular dichroism spectroscopy studies have shown that the modifications do not alter the spatial structure of the peptide. At the same time, succinylation significantly diminishes its bactericidal and cytotoxic potential. We demonstrate that the modification of the bacteriocin is an effect of non-enzymatic reaction with a highly reactive intracellular metabolite, i.e., succinyl-coenzyme A. The production of succinylated forms of the bacteriocin depends on environmental factors and on the access of bacteria to nutrients. Our study indicates that the production of succinylated forms of bacteriocin occurs in response to the changing environment, protects producer cells against the autotoxicity of the excreted peptide, and limits the pathogenicity of the strain.

## 1. Introduction

Succinylation is a posttranslational modification based on attachment of the succinic group (3-carboxypropanoyl, -CO-CH_2_-CH_2_-CO_2_H) to the ε-amino group of lysine residues in proteins. The modification was first described as late as in 2011 [1] and due to its widespread distribution in different proteomes, it is attracting increasing attention [2,3,4,5,6,7]. Succinylation changes the positive charge of the lysine side chain into a negative one. The change radically alters the overall chemical reactivity and introduces a relatively bulky substituent (+100.02 Da mass shift). The side chain of lysine with its primary ε-amino group constitutes reactive centres in a number of enzymes and is involved in many intra- and intermolecular noncovalent interactions. Consequently, succinylation induces significant changes in the function, catalytic properties, structure, and immunogenicity of proteins and influences many different metabolic pathways. Proteomic studies indicate that succinylation is relatively widespread in nature. For example, it involves 738 different proteins in human HeLa cells, 844 proteins in human gastric cancer cells, 861 proteins in mouse liver, 184 proteins in mouse heart, or 990 proteins in *E. coli* [5,7].

Succinylation occurs predominantly via a nonenzymatic mechanism where proteins are modified by succinyl coenzyme A (suc-CoA), a common metabolite in the tricarboxylic acid (TCA) cycle. Suc-CoA is a highly reactive thioester, and its reactivity is a consequence of the ability to form cyclic succinic anhydride intermediate reacting with primary amines in a dose- and pH-dependent manner [8]. Effective succinylation of proteins by suc-CoA is a further consequence of the high level of this metabolite in cells (100–600 nM in eukaryotic mitochondria and ca. 100 nmol g^−1^ in bacterial cells) [7,9]. The level of succinylated proteins in certain cells results from the equilibrium between the activity of enzymes catalysing suc-CoA formation and enzymes that desuccinylate proteins. The former group of enzymes is represented by a critical constituent of the TCA cycle—an α-ketoglutarate dehydrogenase complex (KGDHC) and, to a lesser degree, succinyl-CoA ligase (or succinyl-CoA synthetase, SCS) as well as carnitine palmitoyltransferase 1A (CPT1A) [7]. All these enzymes regulate the level of succinylated proteins by maintaining the level of cellular suc-CoA. In turn, the most common enzyme that is able to desuccinylate proteins is Sirtuin 5 (SIRT5), which is active both in bacteria and in eukaryotic cells [10]. The enzyme removes the succinyl group in an NAD^+^-dependent manner, yielding a free lysine side chain, nicotinamide, and succinyl-ADP-ribose [2].

**Figure 1:**
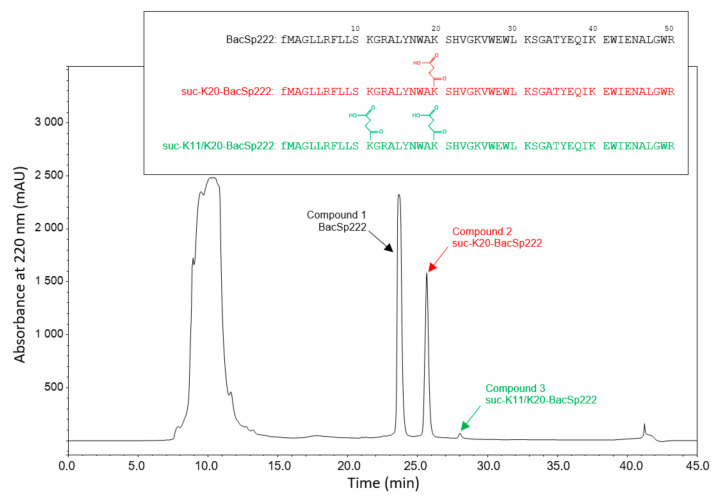
RP-HPLC chromatogram from purification of BacSp222 and its succinylated forms (denoted by black, red and green characters, respectively). The peptide sequence and the succinylation places are presented in the insert. fM denotes the formylated methionine residue at the N-terminus.

A large number of comprehensive studies address the influence of succinylation on the complex proteomes in different cells, tissues, diverse metabolic pathways, diseases, and microorganisms [2,3,5,6,7,11,12,13,14,15,16,17]. At the same time, there are limited numbers of publications that focus on the influence of succinylation on the structure and functions of particular proteins. These studies are primarily focused on evaluation of the effects of in vitro succinylation on such proteins as bovine serum albumin, γ-globulin, lysozyme, and glutamate dehydrogenase [8,18,19]. The influence of physiological succinylation on the structure and activity of a functional protein was most extensively studied in the case of αB-crystallin from the human lens [20]. The present work reports investigation of the structure, biological activity, and mechanism of succinylation of bacteriocin BacSp222. This 50-amino acid long peptide is produced and excreted into the culture medium by a commensal bacterium from dog skin *Staphylococcus pseudintermedius* strain 222 [21]. The peptide is encoded on a 15 kb p222 plasmid, does not contain cysteine, is rich in tryptophan, lysine, and arginine residues, and has formylated methionine at the N-terminus (Figure 1). The sequence of BacSp222 does not exhibit significant similarities to other known proteins. However, our earlier nuclear magnetic resonance (NMR) investigation of its structure documented close similarity of BacSp222 to four bacteriocins: aureocin A53, lacticin Q, entrocin 7A/JSA, and enterocin 7B/JSB. The similarity and the particular structural features of the BacSp222 molecule allowed us to distinguish a new four-helix bundle type of bacteriocins belonging to subclass IId of bacteriocins from Gram-positive bacteria, comprising linear non-pediocin-like peptides [22].

Bacteriocins are defined as ribosomally synthesized peptides or proteins produced by bacteria and capable of killing phylogenetically related strains at very low concentrations [23]. BacSp222 affects a broad range of staphylococci at micromolar concentrations by forming pores in their membranes, most probably via a barrel-stave mechanism [24]. Most interestingly, the biological role of BacSp222 also involves features typical for virulence factors, i.e., a group of molecules produced by pathogenic microorganisms facilitating the replication and dissemination within the host by subverting or eluding defence mechanisms [25]. In particular, BacSp222 is cytotoxic to many eukaryotic cells, including human skin fibroblasts and keratinocytes as well as murine monocytes and macrophages.

Moreover, at low concentrations (in a nM range), BacSp222 efficiently enhances interferon gamma-induced nitric oxide (NO) release in macrophage-like cell lines. In consequence, immunomodulatory activity of the peptide has been suggested, as NO is a multipotent factor inhibiting leukocyte infiltration and proliferation and regulating cytokine expression and cyclooxygenase activity [21]. Other immunomodulatory activities of BacSp222 suggest protein receptor-dependent action, which is presently being investigated in a separate study.

In the present work, we show that BacSp222 is secreted by *Staphylococcus pseudintermedius* 222 in several forms modified by succinylation. A detailed analysis of the structure, biological activity, and mechanism of post-translational modification of these peptides is reported.

## 2. Results and Discussion

### 2.1. Staphylococcus pseudintermedius 222 Produces Native BacSp222 and Its Two Posttranslationally Modified Forms: Suc-K20-BacSp222 and suc-K11/K20-BacSp222

The RP-HPLC separation of the *S. pseudintermedius* 222 post-culture medium revealed the main peak of bacteriocin BacSp222 and two additional compounds (No. 2 and 3; Figure 1, the peaks are indicated by red and green arrows). The corresponding compounds were purified to homogeneity using a second RP-HPLC step. The molecular masses were 5921.9, 6020.3, and 6120.2 Da, respectively, for BacSp222 and compounds No. 2 and 3, showing an equal ca. 99-Da mass increase between each form. The mass spectrometry analyses of tryptic peptide fragments of the compounds of interest revealed modifications in one tryptic peptide (residues 14–25, sequence ALYNWAKSHVGK) in compound 2 and in two tryptic peptides (residues 7–13, sequence FLLSKGR and residues 14–25, sequence ALYNWAKSHVGK) in compound 3. The molecular masses of peptides 7–13 and 14–25 were determined as 919.54 Da and 1472.79 Da. The comparison with the theoretical mass of the respective peptides, i.e., 819.51 Da and 1372.74 Da, indicates a 100-Da increase in each case. The comparison of the MS/MS and MS^3^ spectra confirmed the modification at lysine residues, particularly K5 in peptide FLLSKGR and K7 in peptide ALYNWAKSHVGK. The determined mass shift was used to deduce the possible modification. Based on the annotation from Unimod, an online database of protein modifications for mass spectrometry (http://www.unimod.org/, accessed on 20 May 2016), and Zhinong Zhang et al. [1], two most probable modifications were chosen for further evaluation: succinylation or addition of a methylmalonyl group. Since no significant neutral loss of CO_2_ (−44 Da) for single charged ions nor −22 Da for double-charged ions was observed, as would be expected for methylmalonyllysine, we concluded that succinylation was the modification found in the BacSp222 variants. Overall, the mass spectrometry analyses identified compound 2 as a BacSp222 molecule succinylated at lysine 20 (suc-K20-BacSp222), and compound 3 was identified as a BacSp222 molecule succinylated at lysines 11 and 20 (suc-K11/K20-BacSp222).

These conclusions were independently verified by analysing tryptic peptide fragments of all forms of BacSp222 using automated protein sequencing. The chromatogram corresponding to the sequencing cycle of residue 20 (peptide 14–25 from suc-K20-BacSp222) and from the sequencing cycles corresponding to residues 11 and 20 (peptides 7–13 and 14–25 obtained from suc-K11/K20-BacSp222) revealed an unusual amino acid peak with retention time corresponding to the synthetic N-ε-succinyl lysine standard (Supplementary Materials Figure S1). This finding supports the conclusion that *S. pseudintermedius* 222 produces unmodified native BacSp222 bacteriocin and two succinylated forms: suc-K20-BacSp222 and suc-K11/K20-BacSp222.

Secretion of factors from cells requires transmembrane transport. Bacteria are equipped with a variety of protein secretion systems. Two types dominate the secretion of bacteriocins by Gram-positive bacteria: the general secretion system (Sec) or the Twin-Arginine Transport (Tat) pathway [26]. Both these systems require a secretion signal in the exported molecule, while BacSp222 is not equipped with such a propeptide, as we have shown earlier [21]. Other authors have suggested the involvement of the ABC-type multidrug resistance transporter protein LmrB in the export of such leaderless bacteriocins [27]. In this study, we tested whether the excretion of BacSp222 and its modified forms was related to production of extracellular vesicles. Post-culture medium deprived of bacterial cells was fractionated using a 100-kDa cut-off membrane, and then the BacSp222 forms were determined both in the presumed vesicle-containing fraction and in the low-molecular-weight filtrate. The results clearly show that all BacSp222 forms are contained in the soluble fraction but not in the vesicles. Simultaneously, we could not detect any bacteriocin form in the bacterial cytoplasm, which suggested rapid export of these peptides immediately after synthesis. The mechanisms involved remain to be determined.

Identification of lysines 11 and 20 as sites of succinylation raises a question of why the other lysine residues in the BacSp222 molecule (K25, K31, and K40) do not undergo such modification. Numerous studies mapping thousands of succinylation sites in proteomes of different microorganisms and in animal and human tissues contributed to identification of succinylation patterns and development of machine learning algorithms to predict succinylation sites in particular proteins based on their amino acid sequences, amino acid properties, and theoretical spatial arrangement of amino acid residues [28,29,30,31,32,33,34]. We evaluated the following tools: GPSuc [31], iSuc-PseAAC [34], iSuc-PseOpt [30], pSuc-Lys [29], SuccFind [28], and SuccinSite [32]. However, the results poorly match our experimental findings. Only three algorithms (iSuc-PseOpt, pSuc-Lys, and iSuc-PseAAC) indicated K20 as a potential succinylation site. The most frequently indicated site of succinylation was K30 (indicated by four algorithms), while two other algorithms indicated K40. The inconsistency between the prediction and the experimental data and between the different algorithms demonstrates considerable lack of understanding of the determinants of succinylation site selection. The algorithms clearly require further improvement to take into account the structure of considered proteins and include more information on species-specific factors.

### 2.2. Succinylation Does Not Significantly Influence the Overall Structure of BacSp222

**Figure 2:**
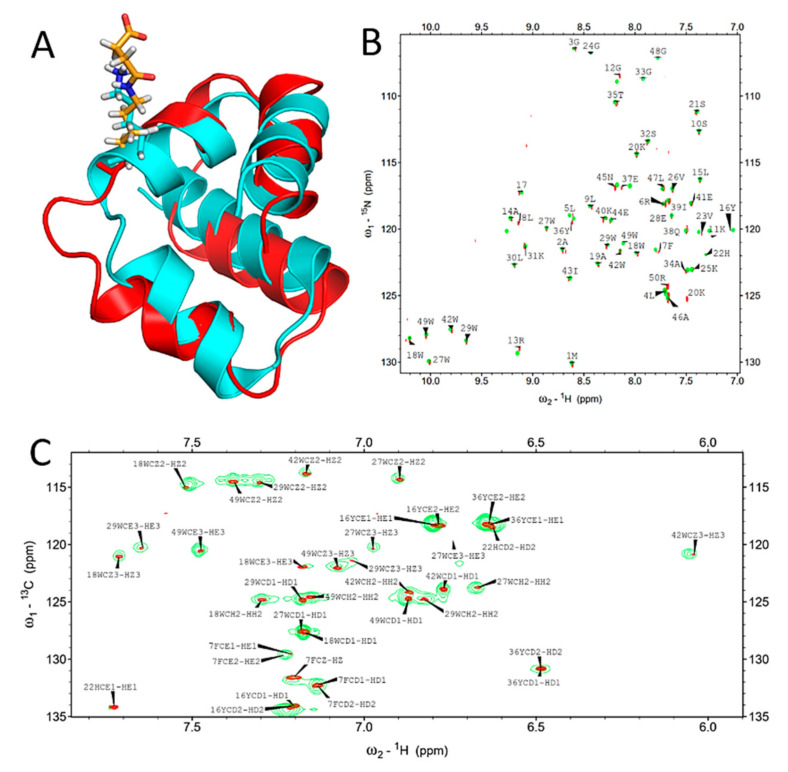
Results of NMR studies of the structure of unmodified and succinylated forms of BacSp222. Panel (**A**) illustrates the superimposition of the most representative structures of unmodified BacSp222 (cyan) and suc-K20-BacSp222 (red). The side chain of lysine 20 in both structures is represented as sticks–hydrogens white, nitrogens blue, carbons of unmodified lysine cyan, and carbons of modified lysine orange. The superimposition of ^1^H-^15^N HSQC spectra and ^1^H-^13^C HSQC spectra tuned to aromatic carbons for unmodified BacSP222 (green) and suc-K20-BacSp222 (red) is presented in panels (**B**),(**C**), respectively.

The influence of succinylation on the bacteriocin structure was investigated using suc-K20-BacSp222 and two complementary techniques-NMR and circular dichroism (CD) spectroscopy. The overlay of the ^1^H-^15^N HSQC spectra of the native and modified BacSp222 (Figure 2B) demonstrates that succinylation of lysine 20 does not significantly influence the backbone chemical shifts, with the exception of L8 and E37. The significant changes in chemical shifts for those residues may not be explained by the proximity of the modification site. The overlay of the ^1^H-^13^C HSQC spectra tuned to aromatic carbons (Figure 2C) demonstrates that resonances characteristic for residues buried in the hydrophobic core of the peptide remain mostly unaffected. The chemical shift assignment of succinylated BacSp222 was based on chemical shifts obtained previously for an unmodified peptide (BMRB accession number 34044) and carefully verified by analysis of a set of 2D homo- and heteronuclear NMR spectra. 80% of chemical shifts were assigned (75% of backbone atoms, 79% of protons and carbons of aliphatic side chains, and 93% of protons, nitrogens, and carbons of aromatic side chains). 

The structure of succinylated BacSp222 calculated form NOEsy restraints was virtually identical to the structure of the unmodified peptide (Figure 2A). The mean global backbone RMSD calculated for the structures of modified and unmodified BacSp222 was 1.9 ± 0.9 Å, indicating no significant structural differences. The statistics of 20 lowest energy structures of modified BacSp222 are presented in Supplementary Materials Table S1.

The comparison of the circular dichroism spectra recorded for BacSp222 and suc-K20-BacSp222 indicated no significant changes. The calculated content of the helical structure within the succinylated form was slightly increased (75% in comparison to 81%, Supplementary Materials Figure S2A), but this difference was within the relatively large uncertainty of such estimations. The CD analysis additionally allowed us to estimate the structural changes in the peptide molecule in the presence of liposomes, which was particularly interesting, as our previous studies revealed that the killing activity of BacSp222 is related to disruption of the bacterial membrane [21]. The spectra of native and succinylated bacteriocins in the presence and absence of liposomes are similar but, again, the calculated content of helical structures in suc-K20-BacSp222 was higher than in BacSp222 (94.4% in comparison to 86.2%; Supplementary Materials Figure S2A). It is also important that the near-UV CD spectroscopy, providing information on the tertiary structure, gave very similar shapes of the spectra (Supplementary Materials Figure S2B), suggesting that succinylation does not affect the overall shape of the bacteriocin molecule, which also confirms the results from NMR spectroscopy.

It can be speculated that the slight but consistent increase in the helical structure content in suc-K20-BacSp222 reflects a more rigid and/or compact structure of the unmodified bacteriocin. This conclusion is, however, not supported by our NMR result. Many authors emphasize that succinylation is an essential post-translational modification that alters e.g., the structure of proteins [2,3,15,35]; unfortunately, there are almost no experimental findings in favour of such a conclusion. Certain reports on in vitro succinylation of bovine serum albumin or glutamate dehydrogenase suggest such a possibility [4]; however, the only study evaluating the influence of physiological succinylation on the structure of a functional protein was focused on αB-crystallin from the human lens [20]. As shown by the results, the modification does not alter the secondary structure of crystallin but mildly perturbates its tertiary and quaternary structure, consequently leading to increased chaperone activity of this protein. Similarly, the influence of succinylation on the structure of BacSp222 in our case is negligible, if any.

### 2.3. Succinylation Decreases the Antibacterial Activity of BacSp222 Bacteriocin

The antibacterial activity of the BacSp222 forms against selected Gram-positive bacteria was compared with the use of the radial diffusion assay. The unmodified form inhibited the growth of each tested bacterial strain, as shown in our previous study [21]. The activity of the succinylated forms was significantly reduced, depending on the degree of succinylation and the bacterial strain. The residual activity of suc-K20-BacSp222 compared to the activity of the unmodified BacSp222 reached 93% against *Micrococcus luteus*, but only 3% against *Staphylococcus intermedius*. The residual bactericidal activity of suc-K11/K20-BacSp222 against *Lactococcus lactis* was estimated at 49%, while the peptide applied at the tested concentration was completely inactive against *Staphylococcus aureus*, *Staphylococcus intermedius*, or *Staphylococcus pseudintermedius* 22221 (unlike BacSp222) (Table 1). These results indicate that succinylation of lysine reduces the antibacterial activity of BacSp222. The observation is in agreement with other reports on succinylated bactericidal peptides. Cochrane and colleagues demonstrated reduced activity of analogues of the lipopeptide antibiotic cerexin with succinylated hydroxylysine residues [36]. Fuchs and others demonstrated an identical effect in the case of entianin bacteriocin succinylated at the tryptophan residue [37]. Unlike BacSp222, the succinylated forms had no antibacterial activity against producer strain Sp222. This allows us to speculate that succinylation provides a mechanism that protects producer cells against the autotoxicity of the secreted peptide. Other authors have suggested a similar functional role of peptides succinylation. For example, Bowers and colleagues suggested that lysine succinylation of thiazolyl peptides may serve a protective function for the producer organism [38].

### 2.4. Succinylation limits BacSp222 Cytotoxic Activity Against Eukaryotic Cells

**Figure 3:**
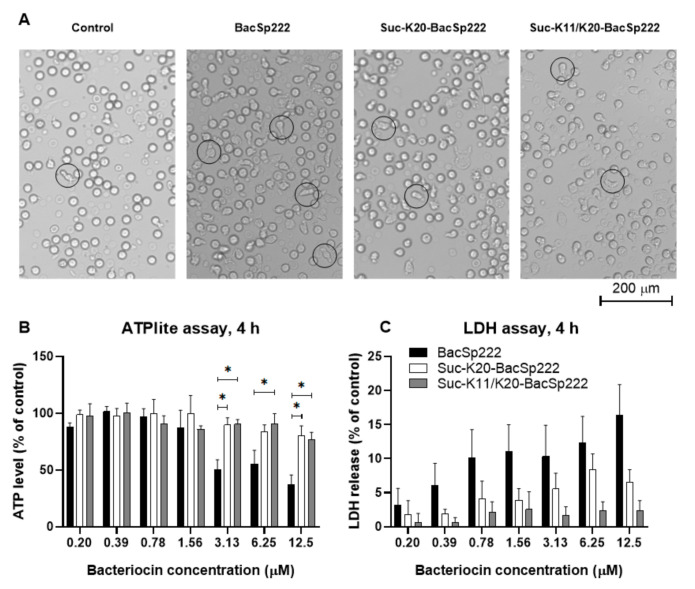
Comparison of the cytotoxic activity of different forms of bacteriocin BacSp222 against human neutrophils. The cells were isolated from healthy volunteers’ blood and immediately exposed to various concentrations of BacSp222 forms for 4 h: unmodified BacSp222, suc-K20-BacSp222, or suc-K11/K20-BacSp222. After incubation, the cells were centrifuged, and the supernatants were transferred to a fresh plate for further analyses. (**A**) Morphological changes were analysed using an inverted light microscope. The circles indicate activated neutrophils. (**B**) The viability of the cells was determined using an ATPlite assay. (**C**) LDH activity in the medium indicates cell membrane damage in the presence of various forms of bacteriocin. * *p* < 0.05.

BacSp222 is a multifunctional peptide with toxicity against both Gram-positive bacteria and eukaryotic cells [21]. Having demonstrated the reduced antibacterial activity of succinylated forms, we wondered whether a similar effect was exerted on cytotoxicity towards eucaryotic cells. The incubation of human neutrophils at 6 µM BacSp222 forms for 2 h induced changes in the morphology of the cells, but most pronounced alterations were observed in cells treated with the unmodified BacSp222. The control (untreated) cells were spherical, whereas a significant number of BacSp222-treated cells were adherent, and their morphology was changed to bipolar. At the same time, only a minor fraction of the cells treated with the succinylated forms exhibited changes in their shape (Figure 3A). Neutrophils can change their shape rapidly, and this phenomenon is associated with migration to sites of inflammation and phagocytosis-dependent killing of microbes [39]. BacSp222 is known to have immunomodulatory properties [21] and such activity results from its interaction with eukaryotic cells. In turn, the immunomodulatory potential of succinylated forms remains to be determined.

**Figure 4:**
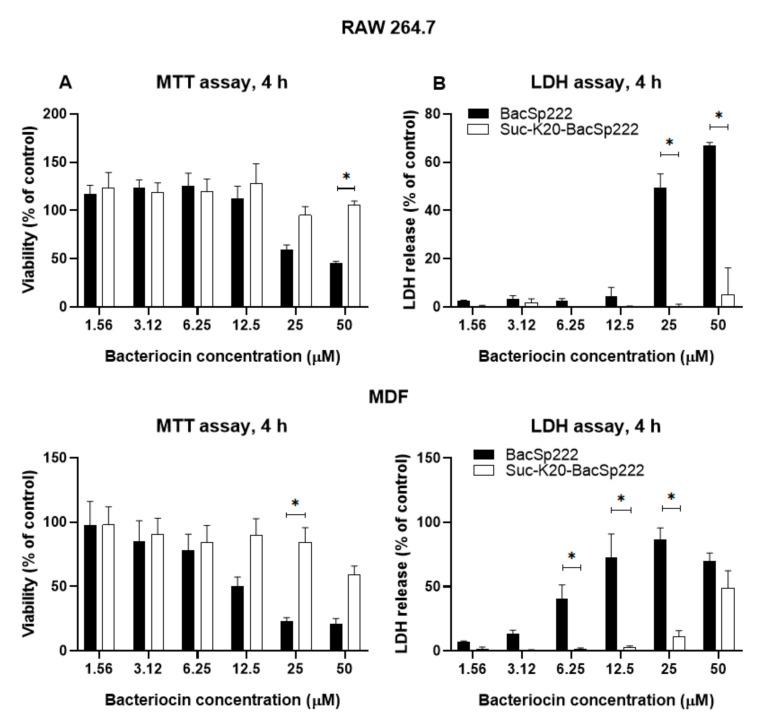
Cytotoxicity of different BacSp222 forms against murine monocyte-macrophage cells RAW 264.7 and murine primary dermal fibroblasts MDF. The cells were incubated for 4 h in a medium containing various concentrations of BacSp222 forms: unmodified BacSp222 and suc-K20-BacSp222. After incubation, the culture media were transferred on a fresh plate for further analyses. (**A**) The viability of the cells was measured using the MTT method. (**B**) The LDH activity in the medium indicates cell membrane damage in the presence of the various forms of bacteriocin. * *p* < 0.05.

We compared the cytotoxicity of the BacSp222 forms considered in this study. The viability of the cells was assessed by determining the level of intracellular ATP (neutrophils) or mitochondrial activity (other tested cells). Additionally, we assessed the integrity of cellular membranes with an LDH release assay. Significant differences between the activities of the BacSp222 forms were observed only at the highest tested concentration. At 3.13 µM, the viability of neutrophils treated with the unmodified peptide was around 50% in the assay conditions, whereas the viability of cells treated with suc-K20-BacSp222 or suc-K20-BacSp222 was about 95% (Figure 3B,C). The reduction in the cytotoxic activity of the succinylated forms, compared to the unmodified bacteriocin, was also observed in the murine and human fibroblasts and in murine monocytic macrophages (Figure 4, Supplementary Materials Figure S3). Only the unmodified form of BacSp222 significantly affected the membrane integrity and viability of the RAW 264.7 and MDF cells (Figure 4). The extension of the incubation time from 4 to 24 h did not significantly affect the results (Supplementary Materials Figure S3). Overall, our results showing the lower cytotoxicity of succinylated forms of BacSp222 agree with literature reports on decreased activity of succinylated variants of other proteins. For example, a decrease in the cytotoxicity of succinylated forms of the EBI protease inhibitor against Molt4 cells was described by Ohba and colleagues [40]. We speculate that the reduced cytotoxic activity and diminished bactericidal effects of the succinylated forms of bacteriocin are a consequence of the modification of the overall charge of the peptide and charge distribution. Introduction of the negative charge is likely to lower the electrostatic affinity of the cationic peptide to negatively charged cell membranes.

### 2.5. BacSp222 Succinylation Is a Nonenzymatic Process Dependent On Suc-CoA and pH, Whereas Desuccinylation Requires NAD^+^ and Cytoplasmic Enzymes

**Figure 5:**
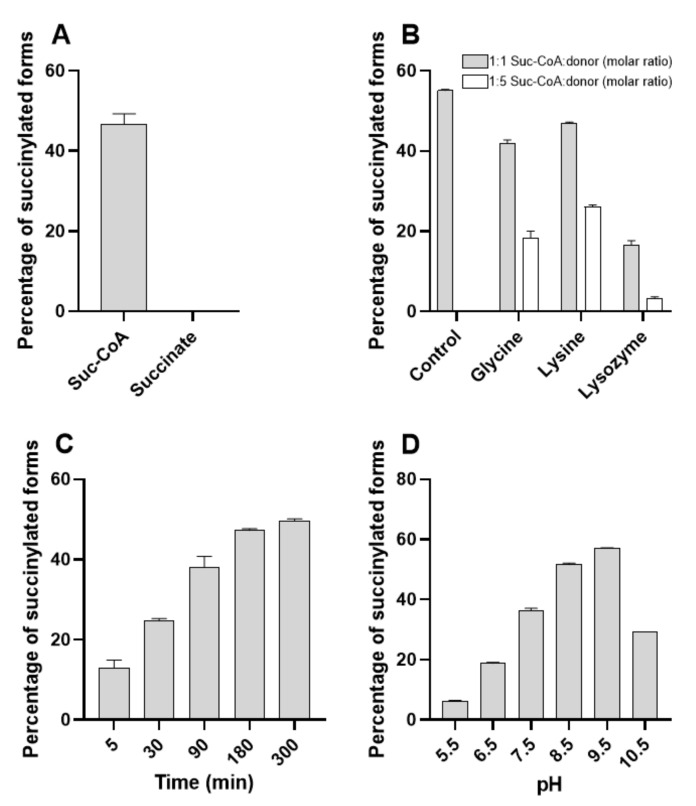
Results of in vitro studies of nonenzymatic succinylation of BacSp222. The unmodified form of BacSp222 was incubated with donors of the butanedioic group in different conditions. Next, the amount of BacSp222 forms was determined by RP-HPLC. (**A**) Ability of physiological donors of the butanedioic group to modify BacSp222. (**B**) Influence of the presence of other molecules containing amino groups on the modification of BacSp222 by suc-CoA. Effect of the reaction time (**C**) and pH of the solution (**D**) on the degree of BacSp222 succinylation.

To establish whether BacSp222 succinylation may proceed via a nonenzymatic reaction, the unmodified bacteriocin was incubated with two physiological donors of the succinyl group existing in living bacteria-succinyl-CoA and succinate. Only succinyl-CoA was able to modify BacSp222 (Figure 5A). The reaction products were essentially identical to products excreted by living bacteria, i.e., suc-K20-BacSp222 and suc-K11/K20-BacSp222. The observed rate of the reaction was relatively fast: after 5 min 13% of bacteriocin was modified (Figure 5C). The reaction diminished gradually over time, and ca. 50% succinylation level was achieved after 180 min. No further increase of the succinylated forms was detected up to 300 min of the reaction. However, addition of a new dose of suc-CoA at 90 min of the reaction increased the level of succinylated forms to 60% (data not shown). These results indicate that the yield of in vitro BacSp222 succinylation is limited by the instability of suc-CoA in the water solution. This explanation is supported by literature data: suc-CoA solutions are stable for about 2 h [41]. The lysine modification reaction is not specific for BacSp222, as equivalent reactions have been described for a variety of proteins [20,42,43]; however, the site-specificity of modification is BacSp222 specific.

The yield of succinylation of BacSp222 by suc-CoA was also dependent on the pH of the reaction. The highest succinylation rate was observed at pH 9.5, whereas the modification of lysines in slightly acidic conditions (pH 5.5) was practically blocked (Figure 5D). This observation agrees with the results of Wagner and Payne, who demonstrated that nonenzymatic BSA succinylation was most efficient at alkaline pH [43]. At pH 9.5, lysine ε-amino groups are significantly protonated, as pKa of this moiety is 10.53 [44]. The most likely mechanism of succinylation comprises the nucleophilic attack of a positively charged ε-amino group of lysine on the carbonyl carbon of the succinyl group in suc-CoA. Additionally, alkaline pH increases the rate of formation of a highly reactive cyclic succinic anhydride intermediate, which reacts with primary amines in a dose-dependent manner [8].

The ability of suc-CoA to react with lysine residues is a common phenomenon [42,43]. Not surprisingly, our further experiments revealed that the excess of non-target amino groups reduces the efficiency of BacSp222 succinylation. For example, a 500-fold molar excess of lysozyme resulted in almost complete inhibition of the BacSp222 succinylation process, decreasing the level of succinylated forms from 55 to 3%. Moreover, the presence of ε-amino groups and α-amino groups affect the succinylation efficiency, as revealed during the reaction in the presence of free amino acids, glycine, and lysine (Figure 5B). Moreover, the presence of the bacterial intracellular or extracellular proteome inhi-bited the succinylation of BacSp222 (data not shown). These results suggest that succinylation of lysine by suc-CoA is nonspecific. The site of modification is most likely dictated by the structural arrangement of the peptide molecule.

We attempted to elucidate the mechanism of possible desuccinylation of BacSp222. Suc-K20-BacSp222 was incubated with bacterial cell lysates and/or with a NAD^+^ solution, and the decrease in succinylated BacSp222 was determined. We showed that bacteriocin desuccinylation was catalysed by constituents of the intracellular proteome of Sp222 and a similar bacterial strain, Sp22221, only in the presence of NAD^+^ (Supplementary Materials Figure S4). A common bacterial NAD^+^-dependent enzyme responsible for the lysine desuccinylation is the sirtuin-like protein CobB [17]. In fact, the *Staphylococcus pseudintermedius* genome contains NAD^+^-dependent protein deacylase [45].

Overall, our results indicate that, contrary to the succinylation process, BacSp222 desuccinylation requires enzymatic catalysis.

### 2.6. Environmental Factors Affect the Level of Excreted Succinylated BacSp222 Forms

**Figure 6:**
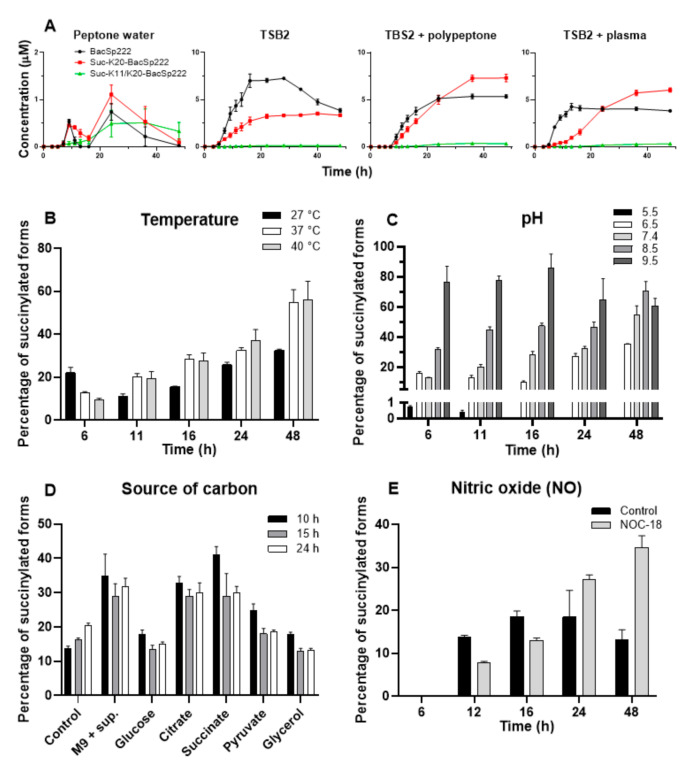
Effect of environmental factors on production of BacSp222 and its post-translationally modified forms by strain Sp222. The bacteria were cultured for various time intervals in different conditions; next, the levels of all BacSp222 forms were determined in the post-culture media by RP-HPLC. (**A**) Effect of the access to nutrients on the biosynthesis of all BacSp222 forms. The successive panels illustrate the effect of culture temperature (**B**), pH of the culture (**C**), carbon source (**D**), and biological stress (presence of nitric oxide) (**E**) on the amount of modified BacSp222forms.

The secretion of all forms of BacSp222 is growth phase-dependent. When bacteria were cultivated in the standard laboratory medium (TSB2), the highest concentrations were detected in the stationary growth phase, i.e., about 7 µM for the unmodified BacSp222, ca. 3.5 µM for suc-K20-BacSp222, and ca. 0.1 µM for suc-K11/K20-BacSp222. The access to various nutrients affected the concentration of the BacSp222 forms produced by Sp222. In the nutrient-poor peptone water medium, the final concentrations of BacSp222 were about seven to five times lower (for the unmodified BacSp222 and suc-K20-BacSp222 forms, respectively), than in the high-nutrient media (Figure 6A). A similar correlation was described by Aasen and colleagues, who demonstrated that the level of sakacin P produced by *Lactobacillus sakei* depended on the availability of amino acids and growth factors [46]. The kinetics of production of different forms was unaffected by nutrient content.

Independently, we wanted to identify specific features that characterize *Staphylococcus pseudintermedius* 222 and ensure BacSp222 succinylation. To this end, we compared the cytosol pH value, the intracellular level of suc-CoA, and the degree of intracellular proteome succinylation between Sp222, two other representative strains of staphylococci (*Staphylococcus pseudintermedius* 22221 and *Staphylococcus aureus* ATCC 25923), and *E. coli* K12. No significant differences in the analysed factors were detected among the different bacteria. *Staphylococcus aureus* was characterized by the highest level of succinylated proteins, which correlated with the highest intracellular pH. All staphylococci were characterized by similar concentrations of suc-CoA, while the Gram-negative bacterium was characterized by a five-fold lower concentration of this metabolite (Supplementary Materials Figure S6). Unfortunately, numerous attempts to produce BacSp222 in a heterologous expression system failed, so we were unable to determine whether the succinylation of the peptide is specific for *S. pseudintermedius* 222. However, the results obtained in this study indicate that the observed succinylation of excreted bacteriocin is not associated with the possible unusual biochemical features of Sp222 in relation to the other bacteria.

We evaluated the influence of the temperature and pH of the medium on succinylation of BacSp222 at different intervals of time during culture (Figure 6B). The lower temperature (27 °C) resulted in a lower percentage of succinylated forms of BacSp222, but this was related to the significantly lower metabolism and bacterial growth rate (two-fold reduction in the optical density of the culture, Supplementary Materials Figure S5B). A high correlation between pH and BacSp222 succinylation was observed. At pH 5.5, the modification of BacSp222 was entirely inhibited, while the overall amount of succinylated forms of BacSp222 at pH 9.5 was greater than 80% (Figure 6C). The observed increase in the succinylated forms at alkaline pH was probably not affected by changes in intracellular pH, as bacteria can maintain the neutral pH of the cytosol even at extreme environmental pH values [47]. However, such maintenance of constant intracellular pH requires a different pattern of expressed proteins and significant changes in the entire metabolic pathways [48]. These overall modifications of cellular physiology are probably the cause of the observed increase in the degree of BacSp222 succinylation.

The level of suc-CoA depends on the activity of the Krebs cycle [49], while the activity of the Krebs cycle is modulated by the availability of particular carbon sources. We assessed whether the level of the BacSp222 forms depends on the type of the carbon source utilised during the bacterial culture. The BacSp222 forms were quantified upon the growth of Sp222 in media containing glucose, citrate, succinate, pyruvate, or glycerol as a primary carbon source and in a control medium without an additional source of carbon at different time points (10, 15, and 24 h) (Figure 6D). The citrate- or succinate-supplemented media were characterized by highest level of succinylated forms, while the lowest level was recorded in those containing glucose and pyruvate. Glucose is known to inhibit Krebs cycle enzymes and stimulate glycolysis. In turn, citrate and other TCA intermediate metabolites activate the Krebs cycle, while pyruvate and glycerol stimulate both glycolysis and the Krebs cycle [50]. We speculate that the high level of modified BacSp222 in samples containing TCA intermediates (citrate or succinate) may exactly be a consequence of the increased intracellular level of suc-CoA.

We have demonstrated that unmodified BacSp222 enhances interferon gamma-dependent NO production in murine macrophages. Nitric oxide is not only a multipotent immunomodulatory molecule but also an effective killing or stressing factor for bacterial cells [21]. We evaluated the effect of NO on the production of BacSp222 forms by Sp222 cells. We applied NOC-18 as a source of nitric oxide [51]. This compound has expected half-life in a solution of ca. 57 h and is able to release nitric oxide steadily. First, we determined that the presence of the bacteria reduced the half-life of NOC-18 (Supplementary Materials Figure S5F), but it was still sufficient for our purpose. The results of the experiment showed that the presence of NO in the bacterial culture enhanced the succinylation of BacSp222 in the late phase of the culture, and the levels of the succinylated forms were twice as high as in the control medium (Figure 6E). Nitric oxide has antibacterial activity [21]; however, the applied concentration of NOC-18 had no significant effect on the growth of bacteria-the growth was slightly diminished only in the initial phase of the culture (Supplementary Materials Figure S5E). Grosser and co-workers have demonstrated that NO induces the expression of nitric oxide resistance proteins in *Staphylococcus aureus* [52]. Possibly, the ability of *Staphylococcus pseudintermedius* to grow in the presence of NO is related to adequate changes in gene expression, which may further contribute to the enhanced level of BacSp222 succinylation. Nevertheless, such a conclusion requires further investigation. The present results indicate that the level of BacSp222 succinylation depends on the presence of a biological stressor, and Sp222 probably modulates the yield of post-translationally modified forms of BacSp222 in response to the host immune response. This phenomenon suggests that increased production of succinylated forms may be a mechanism to avoid the over-activation of the host immune system; however, this hypothesis requires further verification.

## 3. Materials and Methods

### 3.1. Bacterial Strains and Culture Conditions

The following bacterial strains were used in this study: *Staphylococcus pseudintermedius* 222/PCM 2791 (Sp222), *Staphylococcus pseudintermedius* LMG 22221 (Sp22221), *Staphylococcus intermedius* ATCC 29663, *Staphylococcus aureus* ATCC 25923 (Sa25923), *Staphylococcus epidermidis* ATCC 35547, *Bacillus subtilis* ATCC 6633, *Lactococcus lactis* ŁOCK 0871, *Micrococcus luteus* ATCC 4698, and *Escherichia coli* K12/ATCC 10798 (EcK12). All bacteria were cultivated at 37 °C in tryptic soy broth 2 (TSB2, Sigma, St. Louis, MO, USA), except *E. coli*, which was cultured in Luria-Bertani (LB) broth (Sigma, St. Louis, MO, USA).

### 3.2. Eukaryotic Cells and Culture Conditions

Human blood (containing citrate as an anticoagulant) from healthy volunteers was obtained from the Regional Centre of Blood Donation and Treatment (RCBDT) in Kraków, Poland. RCBDT deidentified the samples, which was relevant in order to keep the subjects’ identity confidential, and the study complied with appropriate rules of exclusion of human subjects. Neutrophils were isolated from the blood using centrifugation in a Ficoll-Paque Plus (GE Healthcare, Chicago, IL, USA) density gradient, and erythrocytes were eliminated by polyvinyl alcohol (POCH, Gliwice, Poland) treatment and osmotic lysis. Murine monocyte/macrophage RAW264.7 cells (ATCC TIB-71), murine monocyte/macrophage P388.D1 cells (ATCC CCL-46), and human skin fibroblast (HSF) cells (ATCC CRL-2522) were obtained from the American Type Culture Collection (Manassas, VA, USA). Murine primary dermal fibroblasts (MDF) were a kind gift from Dr. Krystyna Stalińska (Faculty of Biochemistry, Biophysics and Biotechnology, Jagiellonian University in Kraków). All cells were cultured at 5% CO_2_, 37 °C, and >95% humidity in DMEM (GIBCO, Paislay, UK) containing 5% (*v*/*v*) foetal bovine serum (FBS, GIBCO, Paislay, UK) and 4.5 g glucose/l, except neutrophils, which were cultured in 10% (*v*/*v*) FBS in RPMI medium (LONZA, Basel, Switzerland).

### 3.3. Isolation of BacSp222 and Its Succinylated Forms

BacSp222 and its succinylated forms were purified from *S. pseudintermedius* 222 culture supernatant after 24 h of cultivation in TSB2 at 37 °C with shaking (200 rpm). After centrifugation (5000× *g* for 15 min at 4 °C), the supernatant was cooled to 4 °C and precipitated with ammonium sulphate to 60% saturation for four hours in an ice bath. The precipitated material was recovered by centrifugation at 21,000× *g* for 30 min at 4 °C. The pellet was dissolved in H_2_O, acidified to pH 3.0 with trifluoroacetic acid (TFA), centrifuged again, filtered through a 0.45 μm filter, and subjected to reverse-phase high-pressure liquid chromatography (RP-HPLC) on a Discovery Bio Wide Pore C8 250 × 10 mm column (Sigma, St. Louis, MO, USA). The separations were performed at room temperature using an UltiMate 3000 HPLC system (Thermo, Waltham, MA, USA) and spectrophotometric detection at 220 nm. Two solvents were used: A—0.1% TFA (*v*/*v*) in water and B—0.07% TFA in 80% acetonitrile (both *v*/*v*). After equilibration of the column at 60% of buffer B, a linear gradient from 60 to 100% of buffer B was applied over 20 min at a flow rate of 1.5 mL/min. Fractions containing BacSp222, suc-K20-BacSp222, and suc-K11/K20-BacSp222 were collected, dried in a centrifugal evaporator, dissolved in water, and subjected to the second RP-HPLC step in identical conditions as above. The preparations of peptides obtained had a purity of over 99%, as evaluated by analytical RP-HPLC chromatography. Each batch of purified bacteriocin was essentially free of lipopolysaccharide (LPS), as assayed by an E-TOXATE kit (Sigma, St. Louis, MO, USA).

### 3.4. NMR Spectroscopy and Structural Calculations

The nuclear magnetic resonance (NMR) spectra were acquired using a 0.5 mM solution of native unlabelled suc-K20-BacSp222 dissolved in 650 µL of an H_2_O:D_2_O mixture (9:1, *v*/*v*) containing 100 mM of deuterated sodium acetate, pH 5.0. The NMR experiments were performed using a Varian 700 MHz DDR2 spectrometer equipped with an HCN probe at 25 °C (calibrated with a standard ethylene glycol reference sample). The chemical shifts in the ^1^H NMR spectra were reported with respect to external DSS-d4. The chemical shifts of the ^13^C and ^15^N signals were referenced indirectly using frequency ratios of 0.251449530 for ^13^C/^1^H and 0.101329118 for ^15^N/^1^H [53]. The assignment of the chemical shifts was based on our previous study of an unmodified BacSp222 peptide [22] and verified by analysis of a standard set of 2D NMR experiments consisting of TOCSY [54], NOESY [55], ^1^H-^15^N HSQC, and two ^1^H-^13^C HSQC [56] (the ^13^C HSQC spectra were recorded with the offset, spectral widths, and ^13^C–^1^H coupling constant adjusted to aliphatic or aromatic carbons, respectively). The experimental data were processed using the NMRPipe software package [57], while the processed spectra were analysed with SPARKY software [58]. Relevant parameters of the NMR spectra are provided in Supplementary Materials Table S1. The ^1^H, ^13^C, and ^15^N resonance assignments were deposited in BioMagResBank (BMRB) under accession code 34614.

Distance constraints were derived from the 2D NOESY spectrum. Initial structural calculations were performed with CYANA 3.98 software [59], and the automatic NOESY assignment procedure provided 235 distance constraints. The additional restraints for the backbone φ and ψ angles for regions with a well-defined secondary structure (alpha-helices) were derived from the chemical shifts using the TALOS-N server [60]. XPLOR-NIH 2.26 was used for structural calculations [61]. The topology of two additional amino acids (formylated methionine at the N-terminus and succinylated lysine) was manually added to the XPLOR-NIH 2.26 topology file. Evaluation of the suc-K20-BacSp222 final structures was performed with the Protein Structure Validation Server (PSVS) [62]. Twenty structures with the lowest energy (out of the 400 calculated in the final structural refinement) were deposited in PDB under accession code 7NYI.

### 3.5. CD Spectroscopy

The circular dichroism (CD) spectra were obtained using 0.1 (for the 190–240 nm range) or 0.5 (for the 240–350 nm range) mg/mL solutions of BacSp222 or suc-K20-BacSp222 dissolved in 50 mM sodium phosphate pH 6.0. All measurements were made in a 1 mm quartz cuvette using a J-715 spectropolarimeter (Jasco, Tokyo, Japan) at 25 °C without or in the presence of unilamellar dipalmitoylphosphatidyl glycerol (DPPG, Sigma, St. Louis, MO, USA) liposome suspension. The liposomes were prepared using an Avanti Polar Lipids syringe extruder equipped with a 100 nm filter and mixed with bacteriocins at a 100:1 phospholipid-to-bacteriocin molar ratio. All spectra were recorded with appropriate blank subtractions and averaged from three independent measurements. The amounts of structural motifs were calculated using the BeStSel server [63,64].

### 3.6. Biochemical Techniques

The N-terminal amino acid sequences were determined using automatic protein sequencers Procise 491 (Applied Biosystems, Fosters City, CA, USA) and PPSQ-31A (Shimadzu, Kyoto, Japan). The concentration of purified bacteriocin and its succinylated forms was determined by amino acid analysis as described previously [65], while the concentration of these peptides in mixtures or biological fluids was determined by analytical RP-HPLC using a Discovery Bio Wide Pore C18 250 × 4.6 mm column (Sigma, St. Louis, MO, USA) and a linear gradient 0–100% of buffer B for 15 min developed at 40 °C. The other details of the HPLC separations were identical to those described in Section 3.3. The system was calibrated using a set of standard solutions of the bacteriocin and its succinylated forms.

Digestion of BacSp222 and its succinylated forms by trypsin (Gold grade, Pierce, Rockford, IL, USA) was performed for 17 h at 37 °C in 0.2 M Tris-HCl, pH 8.1, containing 4% (*w*/*v*) sodium deoxycholate, using a 1:20 enzyme-to-substrate mass ratio. To denature the peptides and facilitate digestion, the reaction mixtures were heated to 99 °C for 5 min and chilled. Trypsin was added after chilling. The peptide fragments were purified by RP-HPLC using a Discovery Bio Wide Pore C18 4.6 × 250 mm column (Sigma, St. Louis, MO, USA) and a linear gradient 0–100% of buffer B for 30 min developed at room temperature. The other details of the HPLC separations were identical to those described in Section 3.3.

A synthetic peptide SRVLDGLVMTTISSSK was used to obtain the peptide standard containing N-ε-succinyl-lysine. A quantity of 10 nmol of the peptide was dissolved in 1 mL of 0.2 M sodium borate, pH 8.2, and 3 mg of solid succinic anhydride (Sigma) was added. The reaction was performed at room temperature for 1 h on a magnetic stirrer and at constant pH maintained by dropwise addition of a 5 M NaOH solution. After the reaction, the peptide was digested by cyanogen bromide to obtain the peptide fragment TTISSSL-suc-K, which was used to calibrate the automatic protein sequencer.

For mass spectrometry, the peptides were dissolved in 30% methanol with 0.1% formic acid (both *v*/*v*). An HTC Ultra ETD II mass spectrometer equipped with an electrospray ionization ion source and an electron-transfer dissociation II fragmentation module (Bruker) was used. The samples were injected directly using a syringe pump at a flow rate of 180 μL/hour. The analyses were performed in a positive ion mode with a capillary voltage of 3.5 kV, a nebulizer pressure of 10 psi, a drying gas flow of 5 Lmin, and an ion source temperature of 300 °C. The spectra were acquired in the MS and MS^n^ modes in the range of 100–3000 *m*/*z* with both CID (collision-induced dissociation) and ETD (electron-transfer dissociation) ion fragmentation. The tandem MS data were processed manually using DataAnalysisTM 4.0 and BiotoolsTM 3.2 software (Bruker, Billerica, MA, USA).

To determine the physicochemical character of the excreted forms of bacteriocin, 10 mL of the bacterial post-culture medium was prepared by centrifugation at 10,000× *g* for 20 min to remove bacterial cells. The supernatant was fractionated using a 100-kDa cut-off membrane (Millipore, Burlington, MA, USA). The filtrate and the supernatant containing possible macromolecular aggregates or membrane vesicles excreted by bacterial cells were solubilized with 6 M guanidine hydrochloride (Sigma). The level of each form of bacteriocin was determined by RP-HPLC using a Discovery Bio Wide Pore C8 250 × 4.6 mm column (Sigma) developed in a linear gradient from 0 to 100% of buffer B over 15 min at 40 °C and a flow rate of 1 mL/min. The other details of the separations were identical to those described in Section 3.3.

Bacterial cell lysate was obtained via a combination of enzymatic and mechanical factors. A volume of 10 mL of bacterial culture was centrifuged at 5000× *g* for 10 min. The pellet was washed 3 times wth PBS and suspended in 80 µL of phosphate-buffered saline (PBS, Sigma). The cells were pretreated with 40 µg/mL lysostaphin (Sigma) and 125 U/mL Omni nuclease (Eurx, Gdańsk, Poland) with shaking at 1,000 rpm for 1 h at 37 °C. The suspension was sonicated using a UP50H sonicator (Hielscher, Teltov, Germany, 30 pulses at 100% of power, amplitude 0.5). The incubation and sonication steps were repeated. As a final step, the suspension was centrifuged at 15,000× *g* for 20 min at 4 °C. The collected lysate was stored at −20 °C.

The concentration of succinyl-coenzyme A (suc-CoA) was determined by RP-HPLC using a Discovery C18 250 × 4.6 mm column (Sigma). Two solvents were used: A-150 mM NaH_2_PO_4_ containing 5% methanol (*v*/*v*) and B-150 mM NaH_2_PO_4_ containing 30% methanol (*v*/*v*). After equilibration of the column at 0% of buffer B, a linear gradient from 0 to 100% of buffer B was applied over 7.5 min. The flow rate was 1 mL/min. The spectrophotometric detection was performed at 254 nm while the temperature of the column was maintained at 40 °C. Before measurements, the column was calibrated using a set of standard solutions of suc-CoA (Santa Cruz Biotechnology, Santa Cruz, CA, USA).

The level of lysine succinylation in intracellular bacterial proteomes was determined by Western Blotting. Tris-glycine sodium dodecyl sulphate polyacrylamide gel electrophoresis (SDS-PAGE) was performed in reducing conditions using Mini-Protean Precast Gels (Bio-Rad, Hercules, CA, USA). After electrophoresis, the proteins were transferred on a 0.4 μm pore size polyvinylidene difluoride (PVDF) membrane (Millipore, Burlington, MA, USA) using 10 mM N-cyclohexyl-3-aminopropanesulfonic acid (CAPS) buffer, pH 11.0, containing 10% (*v*/*v*) methanol. The transfer was evaluated by transient staining the membranes using 0.5% (*w*/*v*) Ponceau S dissolved in 5% (*v*/*v*) acetic acid. The immunodetection was performed using a 0.05% (*v*/*v*) solution of anti-succinyl lysine (anti-Ksu) rabbit polyclonal antibodies (PTM BIO, Cesarea, Israel) dissolved in 5% (*w*/*v*) skimmed milk in Tris-buffered saline containing 0.05% Tween-20 (TBST). The primary antibodies were detected using a 0.1% (*v*/*v*) solution of HRP-labelled goat anti-rabbit IgG antibodies (Sigma, St. Louis, MO, USA) in 5% (*w*/*v*) skimmed milk in TBST. DAB peroxidase substrate (Sigma) was used to visualize the results.

Bioinformatic prediction of succinylation sites in BacSp222 was performed using available algorithms: SuccinSite [32], GPSuc [31], iSuc-Opt predictor [30], pSuc-Lys predictor [29], iSucPseAAC [34], and SucFind [28].

### 3.7. Antibacterial Radial Diffusion Assay

A radial diffusion assay was used to compare the bactericidal activity of the BacSp222 forms. Soft TSB plates (0.75% agarose, *w*/*v*) contained the tested bacteria. Aliquots of 5 µL of the tested bacteriocin were added as drops at different sites. BacSp222 was applied in serial two-fold dilutions at concentrations ranging from 0.62 µM to 40 µM, whereas both succinylated forms were used only at the highest concentration (40 µM). After 16 h of incubation at 37 °C, the antibacterial activity of the peptides was determined as the diameter of the bacterial growth inhibition zone. Because the correlation between the diameter of inhibition zones and concentration of the peptides was nonlinear the antibacterial activities of both modified forms of the bacteriocin were calculated and presented as a percentage of the activity of unmodified BacSp222.

### 3.8. Analysis of the Effect of Bacteriocin on Eukaryotic Cells

Human blood neutrophils were transferred to a half-area 96-well plate (50,000 cells per well in 50 µL RPMI, 10% FBS, Sigma, St. Louis, MO, USA). 5 µL of PBS or bacteriocin solutions was added and the cells were cultured for 4 h. After incubation, the cells were centrifuged at 1280× *g*, and the supernatants were used to determine the activity of lactate dehydrogenase (LDH). Cell viability was determined using an ATPlite kit. Both assays were performed in accordance with the manufacturers’ instructions (Roche, Basel, Switzerland, and Perkin Elmer, Waltham, MA, USA, respectively). A positive control for the LDH release was obtained by treating the cells with 5% (*v*/*v*) Triton X-100 (Sigma, St. Louis, MO, USA) in RPMI. The absorbance (in the case of LDH detection) and luminescence (for the viability assay) were measured with the use of a Synergy H1 hybrid plate reader controlled by Gene5 version 2.00.18 Software (BIOTEK Instruments, Winooski, VT, USA). The viability of cells was calculated using the following formula:

(1) $$\mathrm{ATP}\;\mathrm{level}\;\left(\%\;\mathrm{of}\;\mathrm{control}\right)=\frac{\mathrm{Luminescence}\;\mathrm{of}\;\mathrm{bacteriocin}\text{-}\mathrm{treated}\;\mathrm{cells}\;}{\mathrm{Luminescence}\;\mathrm{of}\;\mathrm{PBS}\text{-}\mathrm{treated}\;\mathrm{cells}}\times \;100\%$$

LDH activity was calculated according to the formula:

(2) $$\mathrm{LDH}\;\mathrm{release}\;\left(\%\;\mathrm{of}\;\mathrm{control}\right)=\frac{\mathrm{Absorbance}\;\mathrm{of}\;\mathrm{bacteriocin}\text{-}\mathrm{treated}\;\mathrm{cells}\;}{\mathrm{Absorbance}\;\mathrm{of}\;\mathrm{Triton}\;X\text{-}100\text{-}\mathrm{treated}\;\mathrm{cells}}\times \;100\%$$

The morphology of neutrophils upon the treatment with bacteriocins was inspected using an inverted light microscope DM IL LED Fluo (Leica, Wetzlar, Germany).

RAW264.7, MDF, P388.D1, and HSF cells were seeded on half-area 96-well plates in 50 µL of growth medium at a density of 20,000 cells per well. After 24 h, the media were removed and replaced with fresh DMEM containing 2% FBS and 5 µL of PBS (for control cells) or 5 µL of bacteriocins (final concentrations: 50, 25, 12.5, 6.25, 3.12, and 1.56 µM). After 4- or 24-h exposure to the bacteriocins, the supernatants were collected and used to measure LDH activity (as described above), while the viability of the cells was determined by an MTT assay according to the standard protocol. All measurements were performed using a Synergy H1 hybrid reader running with Gene5 v2.00.18 Software (BIOTEK Instruments, Winooski, VT, USA). The viability of cells was calculated using the following formula:

(3) $$\mathrm{Viability}\;\left(\%\;\mathrm{of}\;\mathrm{control}\right)=\frac{\mathrm{Absorbance}\;\mathrm{of}\;\mathrm{bacteriocin}\text{-}\mathrm{treated}\;\mathrm{cells}\;}{\mathrm{Absorbance}\;\mathrm{of}\;\mathrm{PBS}\text{-}\mathrm{treated}\;\mathrm{cells}}\times \;100\%$$

### 3.9. In Vitro Analysis of Succinylation and Desuccinylation of BacSp222

BacSp222 or suc-K20-BacSp222 were incubated at 37 °C for 3 h (unless otherwise indicated) in PBS or in Britton–Robinson buffer with a 3-carboxypropanoyl group donor such as succinyl coenzyme A sodium salt (Santa Cruz Biotechnology) or disodium succinate (Sigma) at molar ratios of 1:100 (for succinylation studies) or independently with the bacterial lysate or/and with 25 mM NAD^+^ (Roanal) (in case of desuccinylation studies). The pH and ionic strength of the Britton–Robinson buffer were adjusted by KOH and NaCl, respectively. Compounds containing amino groups i.e., glycine (Sigma), lysine (Sigma, St. Louis, MO, USA), and human lysozyme (Sigma, St. Louis, MO, USA) and the BacSp222 variants were used at compound:donor molar ratios of 1:100 or 1:500. The concentration of the BacSp222 forms was determined by RP-HPLC using a Discovery Bio Wide Pore C8 250 × 4.6 mm column (Sigma, St. Louis, MO, USA) and a linear gradient from 0 to 100% of buffer B over 15 min developed at 40 °C and a flow rate of 1 mL/min. The other details of the separations were identical to those described in Section 3.3. The results are presented as a percentage of the succinylated form in the pool of all BacSp222 forms (for succinylation studies) or a percentage of detected suc-K20-BacSp222 relative to the control suc-K20-BacSp222 sample (in the desuccinylation studies).

### 3.10. Analysis of the Influence of Environmental Factors on the Level of Succinylated Forms of BacSp222

*Staphylococcus pseudintermedius* 222 (Sp222) was cultivated in TSB2 or in M9 medium with supplementation dedicated to staphylococci [66] at 37 °C (unless otherwise stated) and 180 rpm shaking for different periods. pH of the media were adjusted with NaOH or HCl. The carbon sources included glucose (BioShop, Burlington, ON, Canada), citrate (POCH), succinate (Merck), pyruvate (Eurochem BGD), or glycerol (BioShop, Burlington, ON, Canada), and were tested at a 60 mM concentration. The donor of nitric oxide, NOC-18 (ChemCruz, Santa Cruz, CA, USA), was used at a 4 µM concentration. Optical density (OD) at 600 nm was used to monitor the growth. After cultivation, the bacterial suspension was centrifuged at 10,000× *g* for 5 min to remove the cells, and the concentration of all BacSp222 forms was determined by RP-HPLC (in conditions as described in Section 3.9.). The results were shown as a percentage of succinylated forms in relation to the amount of all BacSp222 forms.

Nitric oxide release was determined using the Griess assay. Briefly, 50 µL of 1% (*w*/*v*) sulfanilic acid (Sigma, St. Louis, MO, USA) in 2.5% (*v*/*v*) H_3_PO_4_ and 50 µL of 0.1% (*w*/*v*) N-(1-naphtyl) ethylenediamine dihydrochloride (Sigma) in 2.5% (*v*/*v*) H_3_PO_4_ were added to 100 µL of post-culture medium. Absorbance was determined at 545 nm using a Synergy H1 hybrid reader and Gene5 v2.00.18 Software (BIOTEK Instruments, Winooski, VT, USA).

### 3.11. Determination of Intracellular pH in Bacteria

The bacteria were incubated for 30 min at 37 °C with shaking (300 rpm) upon addition of 10 µg/mL of the pH-sensitive dye BCECF-AM (Thermo, Waltham, MA, USA). The cultures were centrifuged for 5 min at 5000× *g*, and the bacterial pellet was suspended at OD of 0.4 in PBS supplemented with 25 mM glucose. The suspensions were mixed with PBS (control sample) or with a solution of Intracellular pH Calibration Buffer (Thermo) containing 10 µM nigericin (Thermo, Waltham, MA, USA) and 10 µM valinomycin (Thermo, Waltham, MA, USA) (for Gram-positive bacteria) or 10 µM CCCP (Sigma, St. Louis, MO, USA) (in the case of Gram-negative bacteria) at a volume ratio 1:1. The mixtures were incubated in darkness at room temperature for 45 min, and the fluorescence at excitation/emission wavelengths of 440/530 nm and 505/530 nm (for Gram-positive bacteria) or 490/530 nm (in the case of Gram-negative bacteria) was determined using a Synergy H1 hybrid reader and with Gene5 version 2.00.18 Software (BIOTEK Instruments, Winooski, VT, USA). The results were analysed using online tool “Quest Graph™ Four Parameter Logistic (4PL) Curve Calculator” [67].

### 3.12. Statistics

Statistical significance for the cytotoxicity assays was calculated by one-way ANOVA on ranks using Statistica v13.3 software (StatSoft), with *p* < 0.05 considered significant. The bars on the charts represent the average values from three experiments  ± SD.

## 4. Conclusions

We have shown that *S. pseudintermedius* 222 excrete bacteriocin BacSp222 in three forms: unmodified and two forms (suc-K20-BacSp222 and suc-K11/K20-BacSp222) modified post-translationally by succinyl groups attached to ε-amino moieties of lysine residues. The NMR and CD experiments have revealed that the structure of suc-K20-BacSp222 does not significantly differ from that of the native unmodified bacteriocin. At the same time, succinylation of BacSp222 leads to a significant reduction in antibacterial and cytotoxic activity. We have also shown that succinylation is a nonenzymatic process with the highest efficiency at slightly alkaline pH, and the Krebs cycle intermediate succinyl-CoA is the most likely physiological donor capable of modifying the bacteriocin. The amount of BacSp222 excreted by bacteria depends on various environmental factors. We demonstrated that the level of BacSp222 forms was associated with access to nutrients and reached the highest values in high-nutrient media. Moreover, the amount of the excreted succinylated forms was higher at a temperature close to that of the host body and in the post-culture media supplemented with citrate or succinate than in those supplemented with glucose. Furthermore, a higher level of succinylated forms of the peptide was excreted when Sp222 was cultured in stress conditions (nitric oxide).

Overall, the reduced bactericidal activity of succinylated forms of bacteriocin interpreted together with the increased amount of succinylated forms in stress conditions suggest that post-translational modifications are a form of protection of producer cells against the secreted bacteriocin. We speculate that (i) the reduced cytotoxic activity of the succinylated forms of the bacteriocin, (ii) the stimulatory effect of NO on succinylation, and (iii) the fact that Sp222 is an opportunistic specie may indicate that the posttranslational modification of BacSp222 is an element of the mechanism responsible for making Sp222 a natural element of the canine bacterial flora rather than a conventional pathogen.

## Figures and Tables

**Table 1:** Antibacterial activities of post-translationally modified forms of BacSp222. The activity of suc-K20-BacSp222 and suc-K11/K20-BacSp222 against selected Gram-positive bacteria was assessed using the radial diffusion assay and expressed as percentages related to the activity of unmodified BacSp222.

Bacteria	Residual Antibacterial Activity in Relation to the Activity of Unmodified BacSp222(Percent ± SD)	
	Suc-K20-BacSp222	Suc-K11/K20-BacSp222
*Bacillus subtilis* ATCC 6633	72.24 ± 4.42	32.66 ± 6.78
*Lactococcus lactis* ŁOCK 0871 strain 239	80.32 ± 3.67	48.95 ± 6.21
*Micrococcus luteus* ATCC 4698	92.97 ± 0.56	41.97 ± 4.77
*Staphylococcus aureus* ATCC 25923	33.20 ± 5.42	N.a.
*Staphylococcus epidermidis* ATCC 35547	64.19 ± 7.80	18.00 ± 7.55
*Staphylococcus intermedius* ATCC 29663	3.24 ± 2.51	N.a.
*Staphylococcus pseudintermedius* 222	N.a.	N.a.
*Staphylococcus pseudintermedius* 22221	49.51 ± 11.44	N.a.

N.a.—not active.

## Data Availability

The study did not report any data.

## Supplementary Materials

The following are available online at https://www.mdpi.com/article/10.3390/ijms22126256/s1, Figure S1: Identification of N-ε-succinyl lysine on a set of three overlayed chromatograms from sequencing cycles of tryptic peptide fragments obtained from succinylated forms of BacSp222; Figure S2: Results of CD measurements in the far (**A**) and near (**B**) UV region for unmodified and succinylated forms of BacSp222 recorded both without and in the presence of DPPG liposomes; Figure S3: Comparison of the cytotoxic activity of different forms of bacteriocin BacSp222 against murine monocyte-macrophage cells (RAW 264.7), murine dermal primary fibroblasts (MDF), human skin primary fibroblasts (HSF), and murine monocyte-macrophage cells (P388.D1); Figure S4: Results of in vitro studies of desuccinylation of suc-K20-BacSp222; Figure S5: Growth curves of Sp222 bacteria showing the effect of (**A**) access to nutrients, (**B**) temperature of bacterial culture, (**C**) pH value of the medium, (**D**) source of carbon, (**E**) presence of nitric oxide; Figure S6: Comparison of selected biochemical factors between *Staphylococcus pseudintermedius* 222 and the other bacteria, i.e., *Staphylococcus pseudintermedius* 22221, *Staphylococcus aureus* 25923, and *Escherichia coli* K12; Table S1: NMR-derived constraints and statistics for the suc-K20-BacSp222 structure calculated with XPLOR-NIH 2.26.
