# Supplementary material for: Structure, Biosynthesis, and Biological Activity of Succinylated Forms of Bacteriocin BacSp222
Source: Int J Mol Sci. 2021 Jun 10;22(12):6256. doi: 10.3390/ijms22126256 (PMC8230399; doi:10.3390/ijms22126256)
Supplement: Supplementary file 1 [file ijms-22-06256-s001.zip › Supplementary Materials Figure S1.pdf]

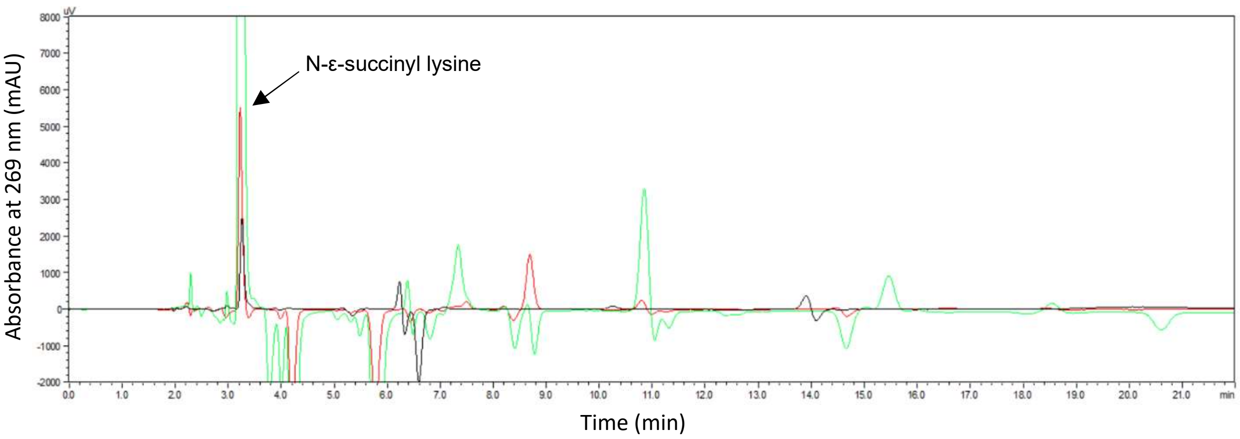

Supplementary Materials Figure S1. Identification of N-ε-succinyl lysine on a set of three overlayed chromatograms from sequencing cycles of tryptic peptide fragments obtained from succinylated forms of BacSp222. The green peak denoted by an arrow shows the synthetic N-ε-succinyl lysine standard. The lower red and black peaks denoted by the same arrow illustrate peaks obtained from sequencing cycles of lysine 11 and lysine 20 residues from posttranslationally modified bacteriocins suc-K20-BacSp222 and suc-K11/K20-BacSp222.
