# Supplementary material for: Structure, Biosynthesis, and Biological Activity of Succinylated Forms of Bacteriocin BacSp222
Source: Int J Mol Sci. 2021 Jun 10;22(12):6256. doi: 10.3390/ijms22126256 (PMC8230399; doi:10.3390/ijms22126256)
Supplement: Supplementary file 1 [file ijms-22-06256-s001.zip › Supplementary Materials Figure S2.pdf]

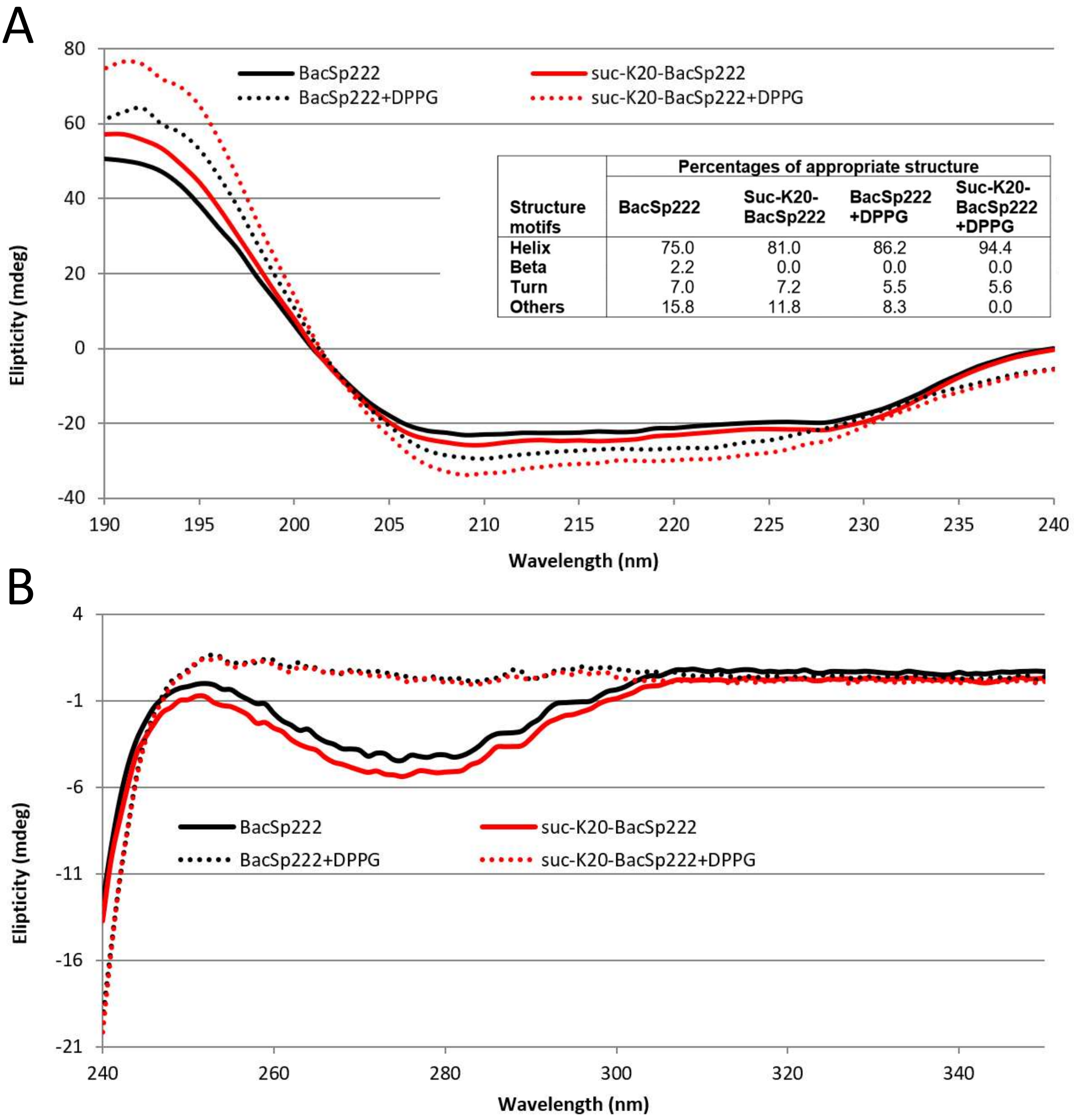

Structure motifs	Percentages of appropriate structure			
	BacSp222	Suc-K20-BacSp222	BacSp222 +DPPG	Suc-K20-BacSp222 +DPPG
Helix	75.0	81.0	86.2	94.4
Beta	2.2	0.0	0.0	0.0
Turn	7.0	7.2	5.5	5.6
Others	15.8	11.8	8.3	0.0

Supplementary Materials Figure S2. Results of CD measurements in the far (A) and near (B) UV region for unmodified and succinylated forms of BacSp222 recorded both without and in the presence of DPPG liposomes. The table inserted in panel A presents the calculated percentage of particular secondary structure motifs.
