# Supplementary material for: Structure, Biosynthesis, and Biological Activity of Succinylated Forms of Bacteriocin BacSp222
Source: Int J Mol Sci. 2021 Jun 10;22(12):6256. doi: 10.3390/ijms22126256 (PMC8230399; doi:10.3390/ijms22126256)
Supplement: Supplementary file 1 [file ijms-22-06256-s001.zip › Supplementary Materials Figure S3.pdf]

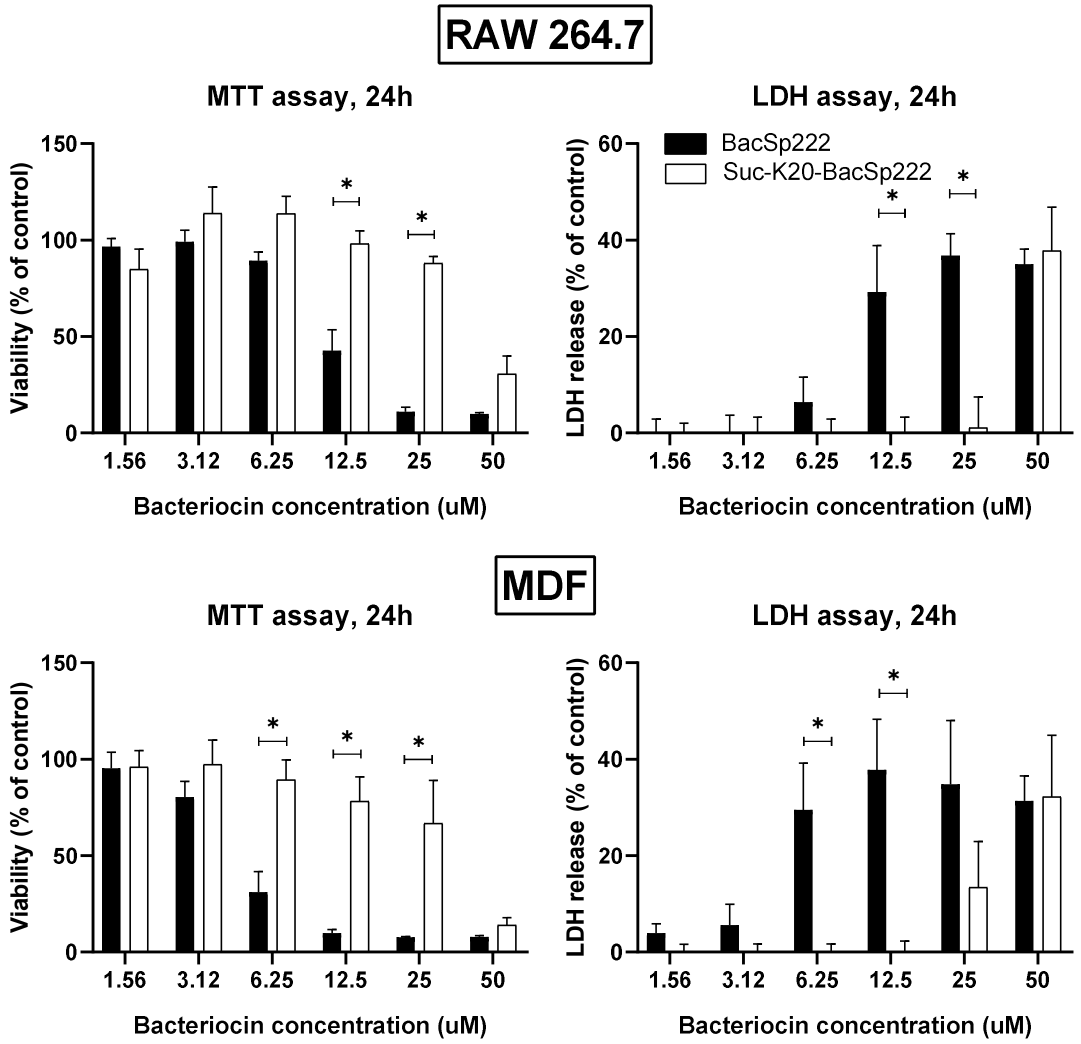

Continued below

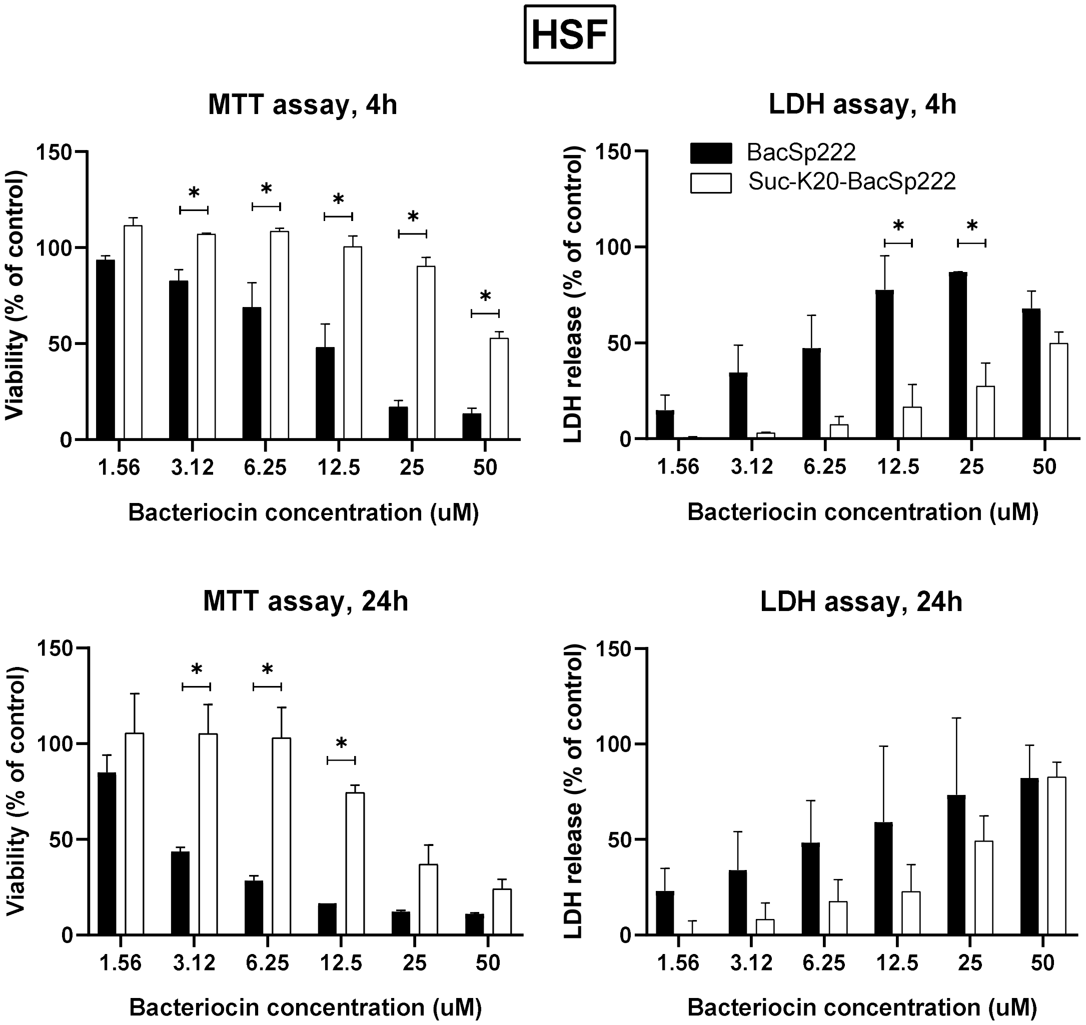

Continued below

P388.D1

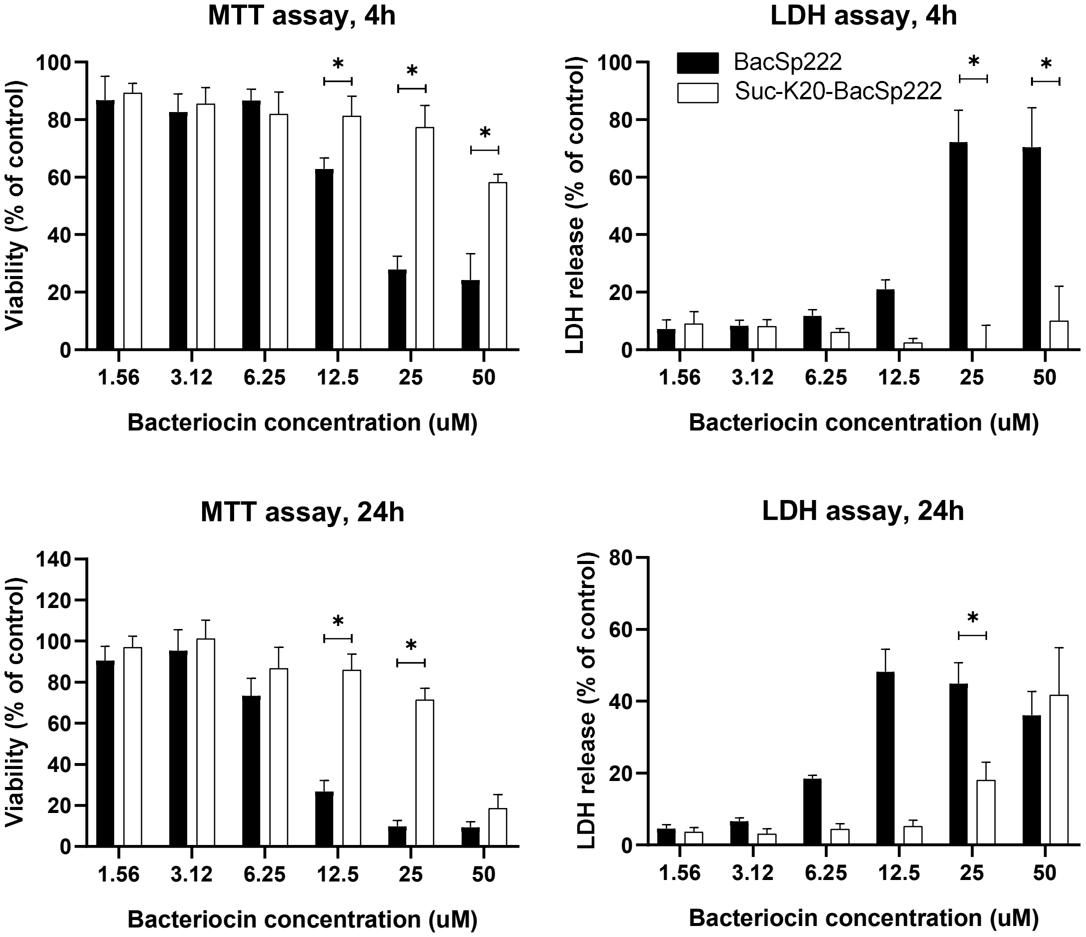

Supplementary Materials Figure S3. Comparison of the cytotoxic activity of different forms of bacteriocin BacSp222 against murine monocyte-macrophage cells (RAW 264.7), murine dermal primary fibroblasts (MDF), human skin primary fibroblasts (HSF), and murine monocyte-macrophage cells (P388.D1). The cells were incubated for 4 or 24 hours at various concentrations of BacSp222 forms: unmodified BacSp222 or suc-K20-BacSp222. After incubation, the culture media were transferred to a fresh plate for further analyses. The viability of the cells was determined using the MTT method. The LDH activity was measured in the medium, indicating cell membrane damage in the presence of the various forms of bacteriocin. *$P < 0.05$.
