# Supplementary material for: Structure, Biosynthesis, and Biological Activity of Succinylated Forms of Bacteriocin BacSp222
Source: Int J Mol Sci. 2021 Jun 10;22(12):6256. doi: 10.3390/ijms22126256 (PMC8230399; doi:10.3390/ijms22126256)
Supplement: Supplementary file 1 [file ijms-22-06256-s001.zip › Supplementary Materials Figure S4.pdf]

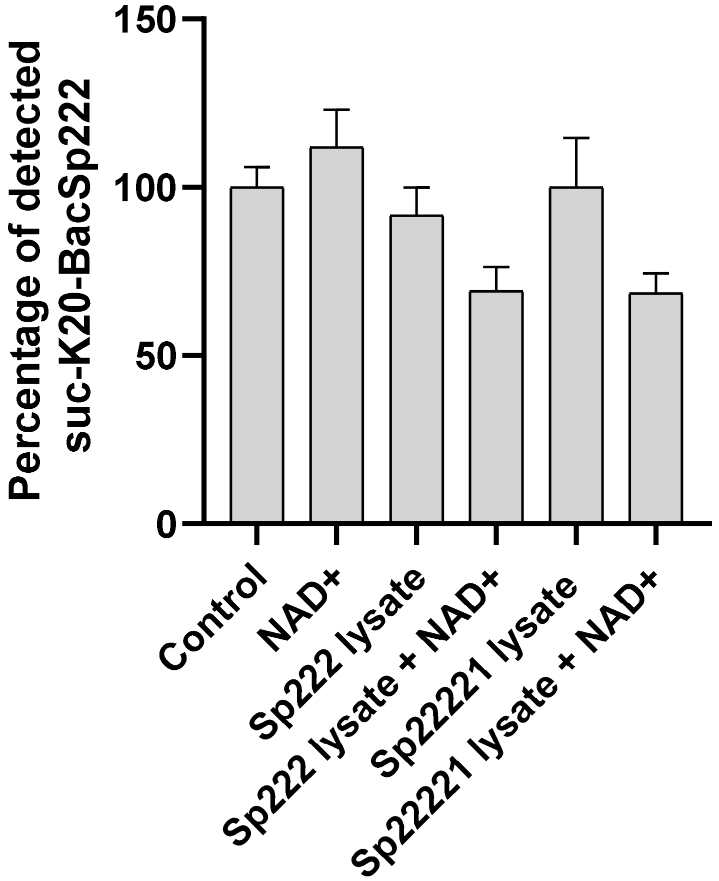

Supplementary Materials Figure S4. Results of in vitro studies of desuccinylation of suc-K20-BacSp222. Suc-K20-BacSp222 was incubated with NAD+ or/and with lysate of bacteria. Next, the amount of residual suc-K20-BacSp222 was determined by RP-HPLC.
