# Supplementary material for: Structure, Biosynthesis, and Biological Activity of Succinylated Forms of Bacteriocin BacSp222
Source: Int J Mol Sci. 2021 Jun 10;22(12):6256. doi: 10.3390/ijms22126256 (PMC8230399; doi:10.3390/ijms22126256)
Supplement: Supplementary file 1 [file ijms-22-06256-s001.zip › Supplementary Materials Figure S5.pdf]

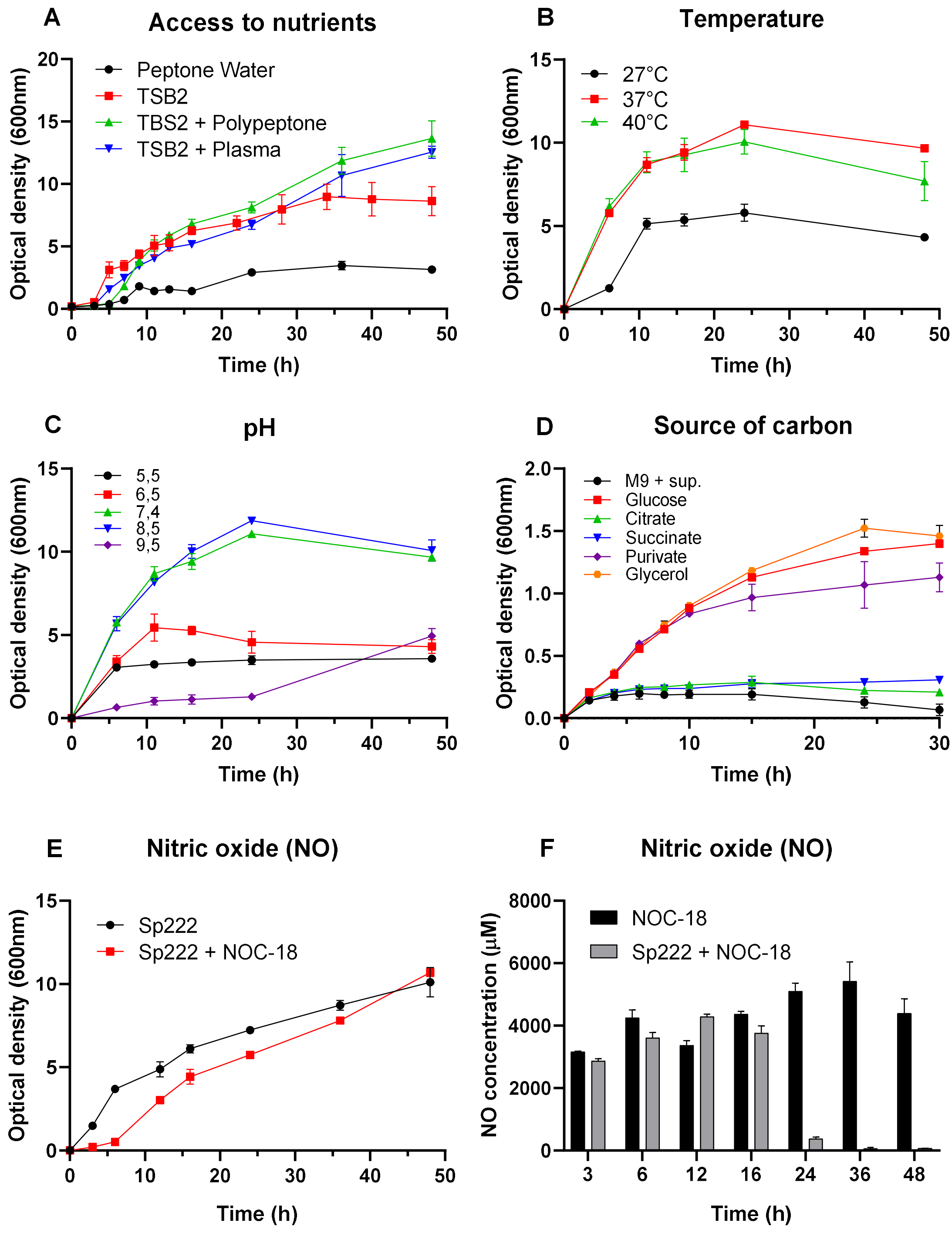

Supplementary Materials Figure S5. Growth curves of Sp222 bacteria showing the effect of (A) access to nutrients, (B) temperature of bacterial culture, (C) pH value of the medium, (D) source of carbon, (E) presence of nitric oxide. (F) Level of nitric oxide released from NOC-18 throughout the experimental period.
