# Supplementary material for: Structure, Biosynthesis, and Biological Activity of Succinylated Forms of Bacteriocin BacSp222
Source: Int J Mol Sci. 2021 Jun 10;22(12):6256. doi: 10.3390/ijms22126256 (PMC8230399; doi:10.3390/ijms22126256)
Supplement: Supplementary file 1 [file ijms-22-06256-s001.zip › Supplementary Materials Figure S6.pdf]

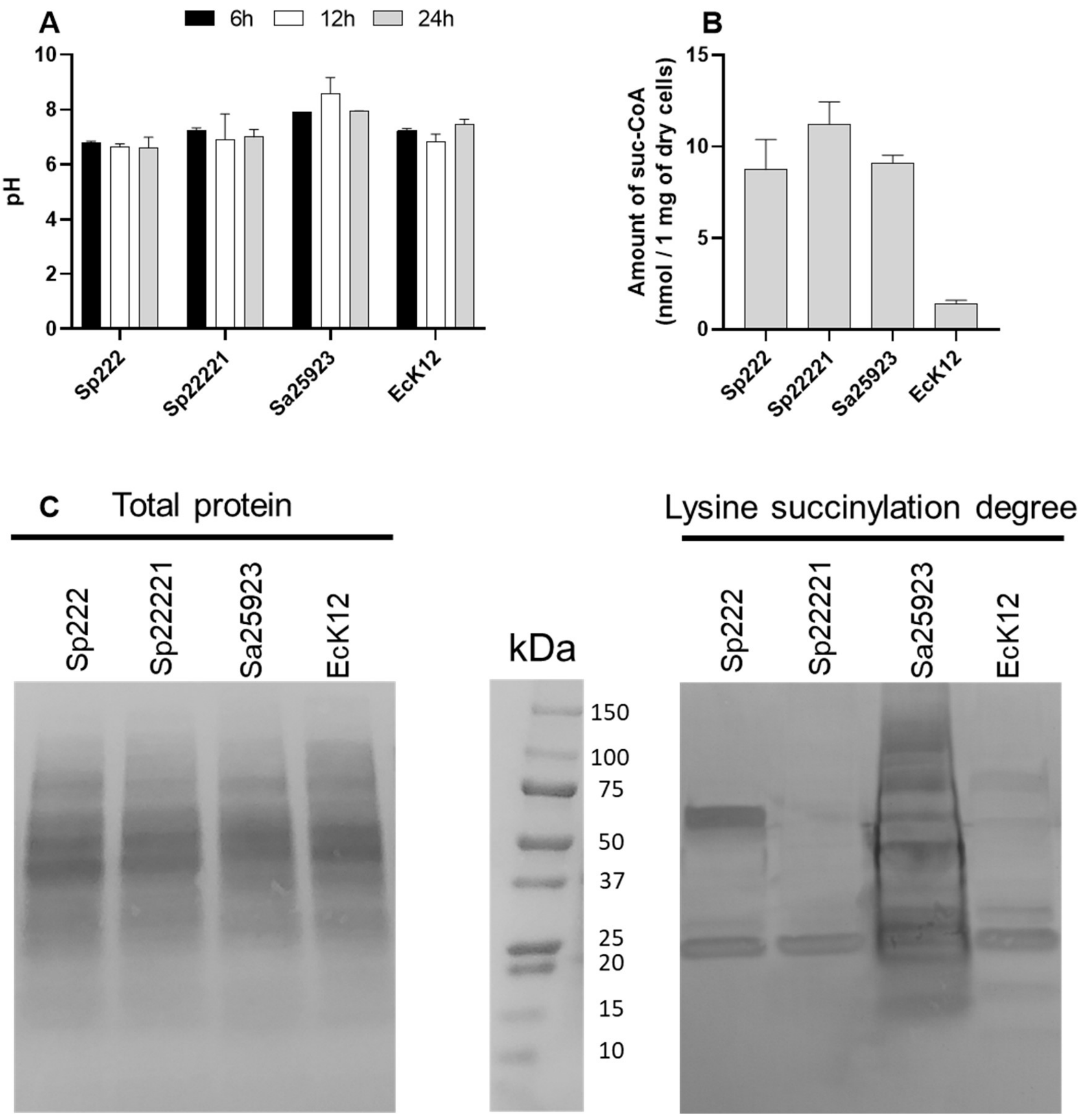


**Supplementary Materials Figure S6.** Comparison of selected biochemical factors between Staphylococcus pseudintermedius 222 and the other bacteria, i.e. Staphylococcus pseudintermedius 22221, Staphylococcus aureus 25923, and Escherichia coli K12. (A) pH values in the bacterial cytosol. (B) Amount of suc-CoA in the bacterial cell lysate determined by RP-HPLC. (C) Degree of succinylation of bacterial intracellular proteins determined with the Western blotting method.
