# Supplementary material for: Structure, Biosynthesis, and Biological Activity of Succinylated Forms of Bacteriocin BacSp222
Source: Int J Mol Sci. 2021 Jun 10;22(12):6256. doi: 10.3390/ijms22126256 (PMC8230399; doi:10.3390/ijms22126256)
Supplement: Supplementary file 1 [file ijms-22-06256-s001.zip › Supplementary Materials Table S1.pdf]

Supplementary Materials Table S1. NMR-derived constraints and statistics for the suc-K20-BacSp222 structure calculated with XPLOR-NIH 2.26.

NOE distance constraints	235
Intraresidual & sequential ($\|i–j\|\leq 1$)	162
Medium-range ($1<\|i–j\|<5$)	23
Long-range ($\|i–j\|\geq 5$)	50
Restraints per residue	6.4
Torsion angle constraints:	
Backbone (φ/ψ)	43/43
Side chains (χ^1/χ^2)	0/0
Deviation from idealized covalent geometry (from PDB validation software)	
Bonds lengths (Å)	0.01
Bond Angles (deg)	1.6
Number of close contacts	1
Ramachandran plot (1..50)	
Residues in the most favored regions (%)	97.5
Residues in additional allowed regions (%)	2.1
Residues in generously allowed regions (%)	0.4
Residues in disallowed regions (%)	0.0
RMSD values	
All backbone atoms	1.1 Å
All heavy atoms	1.6 Å
